# Amorphous carbonized objects and their contribution to reconstructing ancient Mesoamerican cuisine: An innovative non-destructive methodological approach

**DOI:** 10.1371/journal.pone.0334457

**Published:** 2025-11-19

**Authors:** Clarissa Cagnato, Nawa Sugiyama, Laura Longo, Alessandro Bonetto, Matteo Parisatto, Elena Longo, Marko Prasek, Giuliana Tromba, Antonio Marcomini, Elena Badetti

**Affiliations:** 1 Department of Environmental Sciences, Informatics, and Statistics, Ca’ Foscari University of Venice, Mestre, Venice, Italy; 2 Department of Anthropology, University of California-Riverside, Riverside, California, United States of America; 3 Elettra-Sincrotrone Trieste S.C.p.A., Basovizza, Trieste, Italy; Texas A&M University, UNITED STATES OF AMERICA

## Abstract

Archaeobotanists often come across small, amorphous carbonized objects (ACOs) in their flotation samples. Although their identification remains difficult and requires a range of characterization techniques, the study of ACOs recovered from sites in Europe and the Levant have allowed researchers to reconstruct ancient recipes. However, similar materials from sites in pre-Hispanic Central America have been overlooked, hampering our understanding of their ancient cooking traditions. This article proposes a new pipeline to study such remains through three types of non-destructive imaging techniques: optical microscopy (OM), scanning electron microscopy (SEM), and synchrotron radiation-based phase-contrast X-ray computed microtomography (SR micro-CT), key techniques to consider for the imaging of archaeological materials. The approach was developed by establishing a reference collection from modern foods based on traditional ingredients (e.g., maize, manioc) used in the region of interest. This pipeline was then tested on archaeological samples from the ancient site of Teotihuacan (Mexico), which successfully captured the presence of the remains of complex, multi-component food preparations from a feasting context. To the best of our knowledge, this is the first time in which the combination of imaging techniques has been used to discriminate between actual food remains, or simply seeds or plant parts in archaeological contexts from Prehispanic Central America. This study allows to shed more light on ancient recipes and culinary traditions and can be applied more broadly to other contexts in Mesoamerica.

## Introduction

Archaeobotany, through the study of vegetal materials, seeks to study past human-plant interactions. Such studies can provide information on past food production, medicines, fibers, construction materials, but also fuel choices [1]. Plant remains in the archaeological contexts will not preserve equally [2]. Their recovery and preservation will depend on several factors, including the manner in which they enter the archaeological record, the type of plant part discarded, and the environmental conditions of the discard site [3,4]. Moreover, the way a plant is prepared will also play an important role in its preservation in the archaeological record. Plants will survive best in extreme conditions where there is a lack of bacteria, or other destructive agents. Fire, however, can help archaeologists recover ancient plant remains, but this will highly depend on a number of criteria which include the temperature and exposure time [5,6].

Ancient plant remains are most commonly preserved in carbonized form [7–9]. Carbonization, under the right conditions, leads to preservation and can allow for seeds and other plant parts to be fully identified. Using a combination of techniques, which allows for the visualization of both the external and internal properties, finer identifications of seeds and other plant parts have been achieved. Notably, the use of scanning electron microscopy (SEM) has been widely applied in the study of plant remains [10–15]. However, archaeological plant remains may not be recognizable solely based on their external morphological characteristics. Thus, to view the internal structure, it must be decided whether to break the sample (sectioning, microtome) or to apply non-destructive imaging techniques such as X-ray computed microtomography (micro-CT) [16–18], both conventional and synchrotron radiation (SR) based. Applications of this technique, although still underused in the field of archaeobotany, have provided data on the taxonomic identifications of fruits [19], wood [20], parenchyma [21–23], and fibers [24], as well as the identification of domestication syndromes [25–28].

### From sole ingredients to cuisine: previous research on amorphous carbonized objects and their role in understanding identities

Besides the remains of seeds and plant parts that can be more or less identified, in many cases, analysts also might find carbonized remains that lack morphological components even after examination using a stereomicroscope. Over the past decade, scholars have begun to pay closer attention to food remains that do not fit into neat taxonomic categories and to develop new ways to study them, leading, in some cases to the reconstruction of ancient recipes.

From the Early Neolithic site of Tiel Medel in the Netherlands, SEM allowed to determine that wheat (*Triticum* spp.) was the main component of the amorphous objects recovered, while others contained barley [29]. The small size of the pores seemingly indicates a short leavening period leading the author to suggest that these fragments are probably flatbread remains. From Late Neolithic contexts in the Netherlands, “charred isolated lumps of processed plant food” were determined to be the result of cereal-based foods, with in some cases additions of seeds (*Atriplex* spp.; *Bolboschoenus maritimus*) [30]. Arranz-Otaegui et al. [31] used the term “bread-like” to present the remains recovered from Shubayqa 1 in Jordan, a hunter-gatherer site dated to the Natufian period (4.6–11.6 ka cal BP). Their careful analysis revealed that some foods were prepared from only cereals, while others were a mixture of both cereals and noncereal ingredients, likely club-rush (*Bolboschoenus glaucus*) tubers. A large corpus of “cereal fragments” or “agglomerations of fragments” from archaeological sites spanning prehistoric Southeastern Europe were also studied with both a stereomicroscope and SEM [32]. The latter study evidenced the presence of food products that could be separated into two categories: bread/porridge-type foods and ground pounded grains. González Carretero et al. [33] note that “amalgamated plant materials are frequently recovered from Neolithic and later archaeological sites in southwest Asia and Europe”. Their study of lumps of “bread-like” or “porridge-like” remains from Neolithic Çatalhöyük in Turkey were likely the result of breads and/or porridges. Finally, Kabukcu’s et al.’s [34] study of “amorphous, charred plant aggregates” suggests the use of pounded pulses in Paleolithic hunter-gatherer diets. “Bread-like” objects from Neolithic contexts were found to most likely be the result of unleavened cereals that were baked [35]. Malting processes were also identified by Heiss et al. [36] following a study of amorphous charred objects from Predynastic Egypt and Neolithic settlements in Central Europe. The study combined optical microscopy (OM) and SEM to study both archaeological remains and experimentally malted cereal grains. To date however, Heiss et al. [37] are the only researchers to have combined the use of SEM and synchrotron radiation-based phase-contrast X-ray computed microtomography (SR micro-CT) to study “amorphous charred objects”. Their approach allowed them to visualize the dough as well as the pores. Both wheat and barley (*Hordeum* sp.), finely ground, were used as ingredients, while the varying pore sizes and presence or absence of crust indicated different steps in the preparation of the bread. These studies highlight not only what ingredients were used, but also how they combined. This is important as food encodes messages [38–42]. Thus, the culinary transformations of plants (ingredients) into foods can be used to identify and delineate social and cultural identities in the archeological record [43].

In pre-Hispanic contexts, to the best of our knowledge only one example of an amorphous remains has been previously reported. Notably, Capparelli [44] used SEM to study a matrix composed of beans (*Phaseolus* sp.) and chili peppers (*Capsicum* sp.) from the Inca site of El Shincal de Quimivil in Argentina. We are unaware of any similar work involving imaging that also includes micro-CT on amorphous remains on materials from contexts in pre-Hispanic contexts. Therefore, there is a clear need to construct a reference collection and protocol that will permit investigations of complex food preparation strategies, including diverse ways that maize (*Zea mays*) was processed. In the New World, maize/corn was not only the staple food for Mesoamerican societies, but it was also an intricate part of their identity and mythological origin: in fact, they considered themselves people of the corn [45–47]. This plant continues to be a central source of gastronomic diversity in Mesoamerica today, and the myriad of ways in which maize is prepared serves to express and negotiate regional identities [48]. Well-known maize-based foods include tortillas, which are thin, unleavened flatbreads, and tamales, which are made from maize dough filled with meat, fruits, or vegetables, wrapped in leaves, and steamed.

### Teotihuacan as a case study

**Fig 1:**
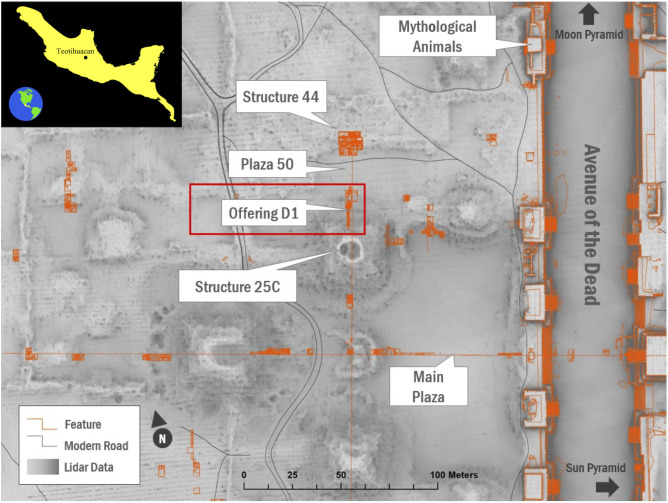
Location of Teotihuacan (inset) and archaeological contexts. Main figure shows the contexts investigated by the PPCC project, and the exact location of Offering D1 (Credit © Project Plaza of the Columns Complex; map insert: modified from © Sémhur/ Wikimedia Commons/ CC-BY-SA-4.0).

The ancient metropolis of Teotihuacan (CE 1–550) is located in the Basin of Mexico in modern-day highland Mexico (Fig 1). The city is located at 2300 m.a.s.l in a semi-arid region, subject to frosts, and a maximum of 800 mm of rainfall during a five-month period. The vegetation was typical of a temperate forest, xerophytic scrub and riparian vegetation, with pine and oak dominating the landscape prior to human settlement [49].

The Project Plaza of the Columns Complex (PPCC) has been excavating a pair of civic-administrative compounds at the core of Teotihuacan since 2015. Preliminary results thus far suggest foreign diplomacy and state-coordinated rituals were essential functions of the complex, with a notable emphasis on Maya high elites, possibly regal individuals visiting or residing long-term at the complex [50,51]. The contexts studied as part of the project are numerous, but for the purposes of this article, we focus on a large burnt cache known as Offering D1 dated to 300–350 CE [50]. This feasting deposit yielded an impressive array of materials, including over 20–25,000 ceramic sherds, + 100,000 faunal and plant remains, across an area of 7 m (east-west) by 2.5 m (north-south). The presence of over 70 fine Maya-style vessels from this context suggests these foreign vessels were likely filled with exotic delicacies and gifted during this extravagant feast. A large diversity of plant taxa has been recovered to date [52,53], indicating the consumption of a variety of local plants, which include maize, opuntia (*Opuntia* sp.), chili peppers, chia (*Salvia* sp.), beans, and amaranth (*Amaranthus* sp.) to name a few. Imported resources such as cacao (*Theobroma cacao*) and manioc (*Manihot esculenta*) were also recovered, the latter evidenced by the presence of starch grains inside a ceramic vessel. Besides the seeds and plant parts that could be identified, an important quantity of ACOs were also recovered in the flotation samples, a process which consists of separating out small, light, buoyant components—i.e., seeds and charcoal—from the rest of the soil. Some ACOs were composed of seeds (whole or fragmented) and while the seeds could be potentially identified, the surrounding matrix could not be solely identified using OM. Overall, the archaeobotanical data from this specific context suggests that participants at the feast consumed foods prepared using local ingredients but also exotic (imported) ones.

Archaeobotanical studies at Teotihuacan have a long history, with multifamily apartment compounds and neighborhood centers especially being well-documented. Studies have shown that the inhabitants had access to a variety of cultivated and wild local plant resources such as maize, squash (*Cucurbita* sp.), chili peppers, beans, chia, amaranth, *Chenopodium*, purslane (*Portulaca* sp.), opuntia, and tomatillos (*Physalis* sp.). Imported products, such as avocado (*Persea americana*), cotton (*Gossypium* sp.), and jocote (*Spondias* sp.) are also documented [54,55]. Combining botanical data from Offering D1 with those previously recovered from Teotihuacan, in addition to what has been reported in other Mesoamerican contexts [52–58], provided a list of ingredients (plants) that could be used in the creation of the experimentally processed (cooked) samples.

## Materials and methods

### Archaeological materials

**Fig 2:**
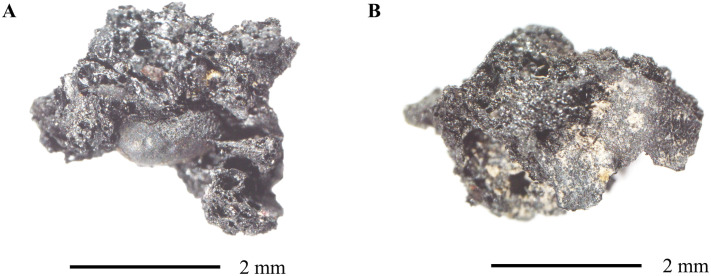
Photographs of the archaeological ACOs from Teotihuacan that were studied for this article. The photographs are from samples MD 4002 (A) and MD 4049 (B).

The ACOs were recovered through the process of flotation. The soil samples collected during the excavation were placed in a bucket with water, and the materials that floted were recovered with fine sieves (0.355 mm) while the heavy fraction was recovered in 1.18 mm and 0.6 mm sieves. The sample was stirred gently in order to break-up the soil matrix, and once all the material had floated, it was placed in fine nylon organza cloth to dry. Once fully dry, they were packed and labeled accordingly. Samples were passed through nested sieves in order to separate them into different size categories. Each size category was individually analyzed. Above 2 mm, all the materials were sorted and weighed. Below 2 mm, only carbonized seeds and plant parts were sorted. Typically, only carbonized materials are considered ancient and therefore included in the total seed count. However, exceptions are made if the materials are semi-carbonized, or when the remain itself is very dense (i.e., nutshell). Material that could not be classified into a neat category was placed into the ACO category. At the laboratory in Teotihuacan, the remains were visualized using an Olympus binocular microscope (10–56×) and photographed using an Olympus U-CMAD3 camera connected to the Olympus DP21 software. In this article, we selected two samples recovered from Offering D1 as examples. The first sample is composed of a seed fragment surrounded in part by amorphous matter (MD 4002), while the second lacks any diagnostic features when viewed under a stereomicroscope (MD 4049) (Fig 2). To carry out further analyses, selected samples were placed in small plastic centrifuge tubes and exported to Ca’ Foscari University (Venice, Italy). All necessary permits were obtained for the described study, namely from the Consejo de Arqueología at the Instituto Nacional de Antropología e Historia (INAH) in Mexico (Oficio 401.3S.16-2022/1004), which complied with all relevant regulations.

### Experimental replicas of foods

**Fig 3:**
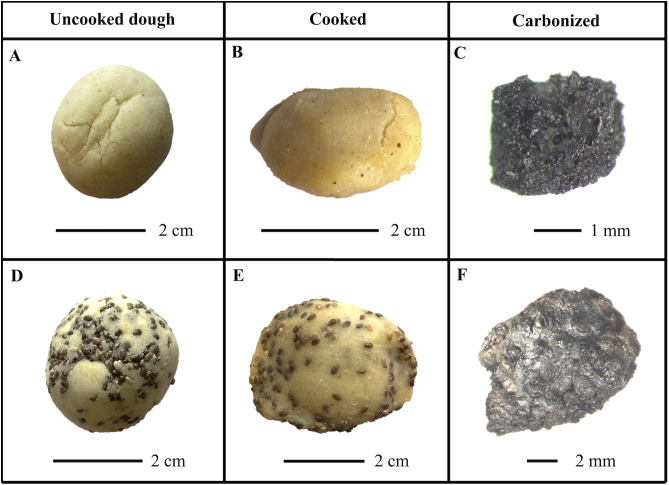
Examples of food replicas shown at different stages of preparation, photos taken using a stereomicroscope. (A-C) Maize tamale; (D-F) Maize tamale with chia seeds.

To develop a reference collection, it was necessary to prepare a series of modern food replicas (Fig 3). In this first instance, we focused on preparing maize-based foods, given the importance of this food at Teotihuacan and throughout Mesoamerica, and others with manioc, a tuber known to have been used by ancient populations [57]. We specifically focused on the preparation of tamales, as a large majority of ACOs from Teotihuacan seem to be multi-ingredient, a feature more characteristic of tamales.

Modern tamales were prepared using two types of flours for the dough (*masa*): maize and manioc. For the maize-based *masa* we opted on using a pre-processed flour that had undergone nixtamalization (Naturelo brand, GMO-free), an ancient process whereby the maize kernels are soaked in an alkaline solution leading to enhanced nutritional properties and facilitating cooking [59], and recently documented in Maya archaeological contexts [60]. The flours were mixed with water and a select number of ingredients, including different-sized seeds such as chia (*Salvia hispanica*) and beans (*Phaseolus vulgaris*). All tamales were prepared using one type of flour for the dough. Although unknown whether fat was an ingredient in the preparation of ancient tamales, we added a vegetable-based butter to one of the doughs. The compositions of each experimental recipe are shown in Table 1.

**Table 1:** Ingredients and quantities in each modern tamale.

Tamale type	Ingredients
Maize (plain)	Nixtamalized maize flour (124 g) with 160 mL water
Maize with vegetable fat	Nixtamalized maize flour (124 g) with 10 g of melted vegetable butter composed of sunflower and rapeseed oil
Maize with chia seeds	Nixtamalized maize flour (124 g) with raw chia seeds (whole)
Maize with beans	Nixtamalized maize flour (124 g) with cooked black beans (whole)
Manioc (plain)	Manioc flour (62g) with 80 mL water

Once the ingredients were added to the *masa*, small balls of around 4–5 cm wide were prepared and wrapped in dried maize husks. To differentiate between the various types of fillings, colored cotton strings were wrapped around each husk. The tamales were then placed in a pot with around 2 cm of water at the bottom, partially covered, and left to steam for around 1 hour. Once cooked, the husks were opened, and the tamales were left to air-dry for a couple of hours. Small pieces (ca. 1–2 cm wide) of each tamale were then taken, taking care that both the *masa* and any included ingredients (i.e., seeds) were present. Samples were then carbonized to approximate the conditions of the archaeological ones (i.e., completely charred/blackened on the outside). Carbonization was undertaken by placing the sample in direct contact with a flame on a methane gas stove, reaching an estimated temperature between 500 and 700 °C. The sample was turned as necessary to ensure that it was evenly carbonized and was kept for at least 10 minutes before removing. Once cool, each sample was individually wrapped in aluminium foil and placed in labeled plastic bags. Carbonized fragments were photographed using a stereomicroscope (8-50x) (Zeiss SteREO connected to a Zeiss Axiocam 208 color camera).

### Imaging

#### Sample preparation.

The archaeological and experimental replicas were studied using different imaging techniques. The first step was to observe them with a stereomicroscope, followed by SEM observations. Finally, a select number of fragments were analyzed using SR micro-CT. The experiments were carried out at the SYRMEP imaging beamline [61] at the Elettra Synchrotron in Trieste, Italy. The ACOs were inserted inside plastic pipette tips (2–200 μl) and opportunely placed to keep the sample stable enough to avoid it from falling. A small quantity of sponge was placed at the larger end to ensure that the archaeological fragment was not damaged if it fell. Finally, the pipette was mounted on the sample holder using beeswax to keep it upright and stable.

#### Data acquisition.

A total of 1800 X-ray projections (i.e., sample radiographs) over a 180° rotation were recorded for each tomographic scan by an Orca Flash 4.0 CMOS camera (2048 × 2048 pixels, with physical pixel size 6.5 μm × 6.5 μm). The detector was coupled with a 17 μm Gadolinium-Gallium Garnet (GGG) scintillator and thanks to a light optical magnification system, the effective pixel size selected for imaging was 0.9 µm x 0.9 µm, leading to a final field of view of 1.8 mm x 1.8 mm. Thus, 1–3 multiple vertical micro-CT steps were required per sample according to the total specimen size. The samples were imaged with a sample-to-detector distance of 150 mm enabling phase-contrast and by using a polychromatic (white) beam filtered by a 1 mm thick silicon layer, resulting in a mean energy of 19 keV. Slice reconstructions, including phase retrieval correction [62], were carried out using the SYRMEP Tomo Project (STP) software [63]. The resulting tomographic images were converted from 32-bit floating point to 16-bit format and cropped, if necessary, in order to significantly reduce the size of the datasets. A combination of various software including the open source ImageJ/Fiji package [64], CT-Analyser (Bruker Corporation) and VGStudioMax (Volume Graphics GmbH) was used for 2D and 3D image processing of tomographic datasets, 3D renderings and dimensional measurements.

#### Image processing.

A customized image processing protocol based on a sequence of grey level thresholding, filtering, despeckling and morphological operations (e.g., 3D closing) was developed to segment the porosity of the doughs and the solid fraction. Volumes of interest (VOIs) of 10^9^ voxels, typically in the form of cubes with a side of 1000 voxels (corresponding to a volume of 0.729 mm^3^) were extracted from each of the considered samples. Only the closed porosity fraction was evaluated here, made up of all those pores completely included within the walls of the dough and not directly connected (at the accessible spatial resolution scale) to the large air bubbles (varying in size from approximately 50 µm to a few hundred µm) entrapped in the dough.

## Results

The observations noted with OM, SEM, and SR micro-CT for each individual type of experimental tamale are presented below. Meaningful features that were observed include the aleurone layer and starchy endosperm in maize-based tamales, the vascular system in manioc-based tamales, and the seed coat and endosperm of chia seeds and bean cotyledons.

### Maize-based tamales (plain and vegetable fat)

The micro-CT reconstructed datasets allowed us to visualize structural information fundamental to identifying the presence of maize. The endosperm of maize kernels is composed of two types of tissue: the aleurone layer and the starchy endosperm; the latter being the largest morphological component of cereal grains [65]. The aleurone layer in maize is described as a single cell layer composed of regular, thick-walled cuboidal cells which, except for the hilar layer at the tip of the kernel, surround the starchy endosperm [66; 67, 68] (see S1 Fig), and are important for the storage of proteins, mineral nutrients, lipids, and vitamins [69]. In our comparative analysis of fresh maize kernels, the aleurone cells were found to be between 25 and 30 μm long and between 15 and 30 μm wide. On average, the cells were larger in flint varieties than in popcorn varieties.

**Fig 4:**
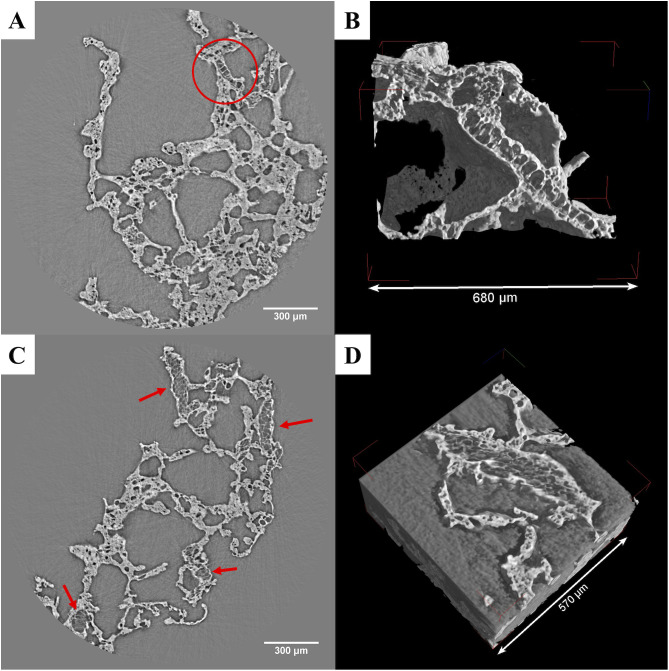
Features observed in the experimental maize-based tamales. The aleurone cells are visible in the plain maize tamale, as seen in 2D (A), and indicated by the red circle, and in 3D (B). Parenchyma cells are visible in the maize tamale made with vegetable fat in 2D (C) and are indicated by the red arrows. D) The cells are also visible in 3D renderings.

In the experimental tamales, this layer was especially striking when viewed in 2D, but it was also clearly visible when viewed in 3D (Fig 4A-B). The aleurone cells we observed were generally rectangular in shape, but in some cases were cuboidal. We observed segments/patches of connected cells in all the maize-based samples, with some having only one segment, with a maximum of 4 segments in the plain maize and maize with vegetable fat samples. Individual aleurone cells varied in size, ranging from 19 μm to 46 μm in length and 13 μm to 37 μm in width, with an average of 33 μm in length and 22 μm in width if we consider all the samples (S1 Table). Using SR micro-CT, the starchy endosperm of maize was also recognized within the matrix of the experimental foods through the presence of patches of parenchyma cells (Fig 4C-D). These thin-walled cells are for storage purposes of starch and proteins [65]. In our samples, these irregular cells ranged between 12 μm and 49 μm, with an average of 22 μm in maximum length. In some cases, the zones of endosperm are clearly associated with patches of aleurone cells, being directly adjacent to the latter. In other cases, the endosperm seems to lack any aleurone cells. We were unable to visualize any starch grains inside these storage cells.

### Manioc tamale

Manioc flour is derived from the tuberous roots of the manioc plant. The tubers are composed of various layers which includes the periderm, followed by the cortex (which includes parenchyma and phloem cells), and then by a large fleshy central core (pith) composed mainly of xylem vessels and longitudinal cells that hold storage parenchyma cells filled with starch grains [70]. Xylem, together with phloem, are the two main parts of a plant’s vascular system. Xylem is important in conducting water and in minor amounts mineral nutrients and other molecules from the roots up to the leaves, while phloem carries sugars [71]. Two types of non-living cells compose primary xylem, notably single-celled tracheids and multi-cellular vessels, the latter which can vary greatly in size [71] and can be also recognized by their wall structure. Vessel elements join to form continuous vessels, and the degradation of their walls creates perforation plates: these can range from simple holes to parallel bars (also known as scalariform). Phloem on the other hand is composed of living cells, known as sieve elements separated by plates [71]. The parenchyma cells in our fresh manioc root varied in size, measuring around 80 μm in length and 20–50 μm in width. Meanwhile, the xylem vessels measured around 80–90 μm in width, with alternate intervessel pits.

**Fig 5:**
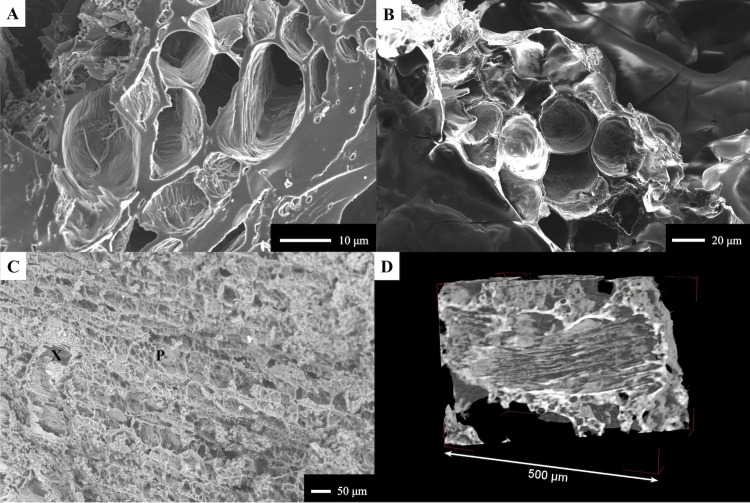
Features observed in the experimental manioc tamale and comparison with modern tuber. Xylem vessels (A) and parenchyma (B), as observed with SEM in experimental tamale; C) modern manioc tuber seen in longitudinal section, imaged with the SEM; X = xylem vessel, P = parenchyma. D) 3D view of experimental tamale possibly showing longitudinal cells (as observed in a modern manioc root- see Fig 5C).

In the experimental tamale, SEM allowed to visualize part of the vascular system of manioc, notably the xylem vessels (Fig 5A). We also observed the presence of empty storage parenchyma cells that measured between 17 μm and 36 μm (Fig 5B), which can also be seen in a modern carbonized manioc tuber (Fig 5C). In the micro-CT images, differentiating between structural elements was not an easy task. We were able to visualize long elongated cells as seen in Fig 5D, which given their size (15–30 μm wide) might belong to the longitudinal cells of the parenchyma found in the pith of the tuber (as seen in Fig 5C).

### Tamale with chia seeds

**Fig 6:**
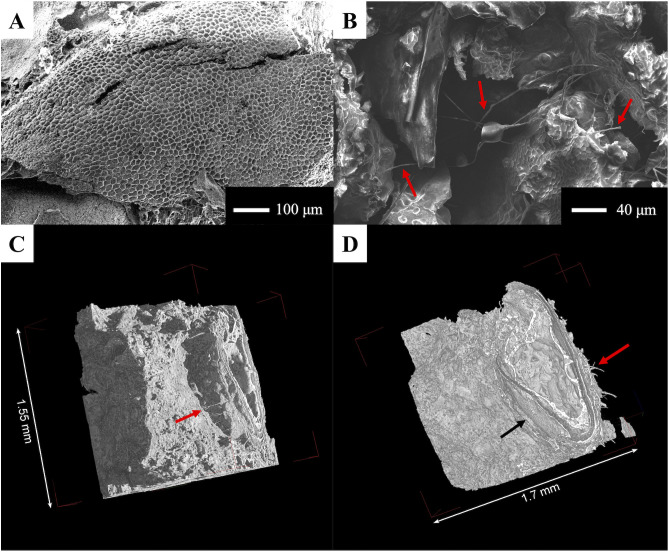
Features observed in the chia seeds embedded in the experimental maize tamale. A) SEM micrograph of the chia epidermis. Mucilage filaments (indicated by red arrows) seen with the SEM (B); and seen in 3D renderings (C-D). The black arrow indicates the epidermis of the seed.

The chia seeds, although having been exposed to fire, could still be recognized with an optical microscope given their shape, size, and outer coat. Fresh chia seeds are composed of three layers. The outer one consists of a regular thin layer (17–18 μm), followed by a thicker and more compact subepidermal layer (40–42 μm). The third and final layer is the endosperm, which at its surface is composed of polygonal isodiametric cells, known as Hex cells [72]. When viewed with the SEM, both the epidermal coat, and the surface of the subepidermal layer (composed of regular cells) were visible. The micro-CT images allowed us to see these three layers very clearly, but this was especially true when viewed in 3D. The subepidermal layer measured 27–34 μm in thickness. The endosperm, especially evident when viewed with SR micro-CT, was absent in many parts, likely due to carbonization. Another feature visible across all imaging types, including micro-CT, was the presence of a fine fibrous network intermixed with the epidermal layer (Fig 6). This feature was visible in both the maize and manioc tamales containing chia seeds. This feature is likely to be mucilage, composed mainly of soluble fiber, which is released by the outer cell wall of the epidermal cells of the chia seed when in contact with water [73–75]. Mucilage is formed very quickly, even after just a few minutes of soaking [76], with the resulting “elongated microfibrils” covering the entire seed [72]. The release of this mucus film leads to disruption of the epidermal cells.

### Tamale with whole beans

**Fig 7:**
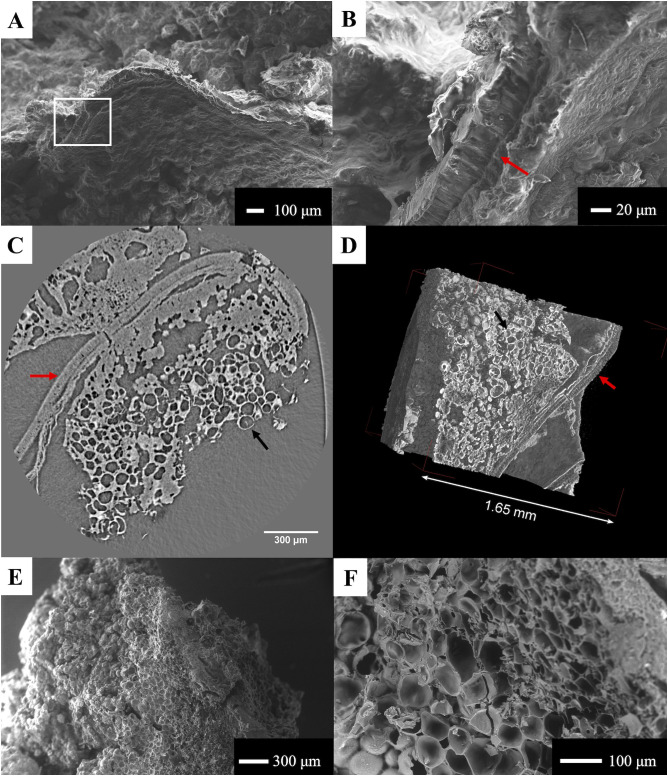
Features observed in the bean cooked in the experimental tamale. Micrographs of the bean seed coat at different magnifications (A-B) with the white rectangle in A corresponding to the area in B, and micro-CT slice (C) and 3D rendering (D) of the bean. The red arrows indicate the palisade cells. The bean endosperm, composed of amyloplasts, can be seen in C-F, as indicated by the black arrows. Empty amyloplasts are clearly visible in C-F.

In the cooked and subsequently carbonized beans, select features were visible both in SEM micrographs but also in the images obtained with SR micro-CT. Beans are composed of the seed coat and the cotyledons. The former is composed of various tissues, with the first being the cuticle, which covers the seed. Below, one can find a layer of palisade cells (also known as macrosclereids), and further below sits a layer of subepidermal pillar cells that contain calcium oxalate crystals. Finally, there is a layer of spongy parenchyma cells before reaching the cotyledon [77,78]. In our samples, we could clearly visualize the macrosclereids, which measured between 46 μm and 55 μm in thickness, with both SEM and SR micro-CT (Fig 7A-D). However, it was difficult to differentiate between the layer of secondary parenchyma cells and the subepidermal layer in 3D. The crystals present in the subepidermal layer are not visible in 3D but they may be represented by the white (denser) spots when viewed in 2D (S2 Fig). A lack of this clear division between the layers may be due to cooking of the bean followed by carbonization of the sample. Perucini-Avendaño et al. [78] found that cooking the bean leads to swelling in the inner part of the seed, with compression/rupture of the seed coat. In the bean cotyledon, we were able to see the parenchyma cells, which measured between 65 μm and 100 μm in maximum length (Fig 7D-F). Parenchyma cells found in the inner testa of legumes (peas, beans, chickpeas, etc.) contain starch grains [79], however, in our experimental sample, these cells appear empty (Fig 7D-F), potentially as a result of the cooking process, which sees the starch moving into the cooking water [78].

### Archaeological samples

#### Sample 1: MD 4002.

**Fig 8:**
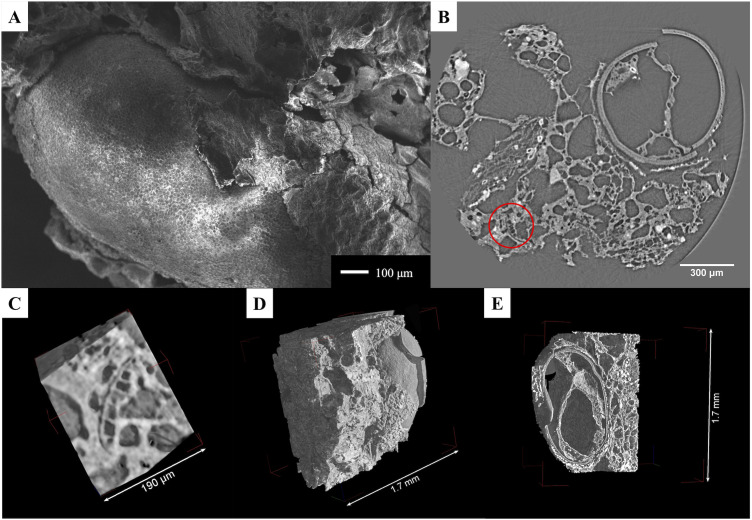
Structural elements observed in archaeological sample MD 4002. A) SEM micrograph of the sample showing the chia seed; B) Micro-CT slice of the sample showing the aleurone layer (inside the red circle); C) The same aleurone layer from (B) rendered in 3D; D-E) 3D renderings of the sample showing the outer (D) and inner (E) portions of the seed and the surrounding matrix.

When viewed under the stereomicroscope, the embedded chia seed was identified based on its general morphology and size. SEM imaging of the seed revealed the surface of the epidermis (Fig 8A), present in some parts, and below, the cells on the surface of the subepidermal layer. The micro-CT slices also showed these same features, with the sub-epidermal layer measuring 24–30 μm in thickness. The internal part of the chia seed (endosperm) was better visualized with SR micro-CT, showing that it was significantly damaged. In terms of the mucilage, we did not observe any with either SEM or with micro-CT.

The tomographic data revealed distinct features within the matrix surrounding the ACO. Notably, four separate segments of a potential aleurone layer were present. These segments were located at 100 μm, 155 μm (two segments), and 715 μm (Fig 8B) from the edge of the chia seed and had cells that measured 22–26 μm in length and 12–24 μm in width. If we consider all four segments, the cells measured on average 23 μm x 18 μm. Moreover, we observed a large mass of endosperm/parenchyma (approx. 735 μm x 215 μm), located at around 210 μm from the chia seed (Fig 8B). Individual cells measured between 15 μm and 36 μm. Another endosperm mass was associated with one of the aleurone patches; the cells measured on average 15 μm.

#### Sample 2: MD 4049.

**Fig 9:**
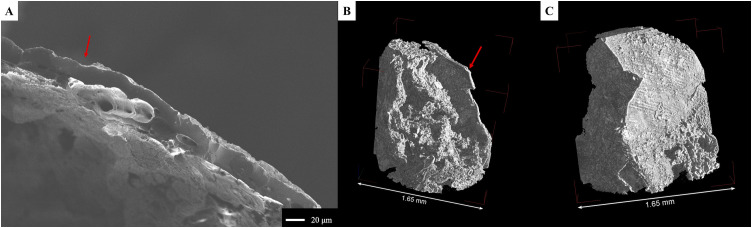
Features observed in the archaeological sample MD 4049. The seed coat is visible in cross-section in the SEM micrograph (A), and in the micro-CT rendering (B), as indicated by the red arrows. The matrix adjacent to the seed coat is visible in (B), while the outer surface is visible in (C).

The stereomicroscope did not reveal any diagnostic features except for the presence of a seed coat. SEM micrographs confirmed the presence of a seed coat with no diagnostic outer structure, but no other clear structures were identified (Fig 9A). The micro-CT 3D datasets of the fragment clearly showed this seed coat (22–40 μm thick), but also revealed the presence of a similar second structure (about 570 μm from the first and 25–33 μm thick), inside the attached matrix (Fig 9B-C). Alongside this latter feature, aleurone cells are visible, measuring 21–28 μm in length and 13–32 μm in width. This combination of elements suggests that at least the inner feature might be a pericarp or testa of a maize kernel.

### Comparison between archaeological and experimental samples

Notable features characteristic of the key ingredients that we have identified include the aleurone cell size, similar to those observed in the maize-based tamales, and the porosity of the matrix/dough.

**Fig 10:**
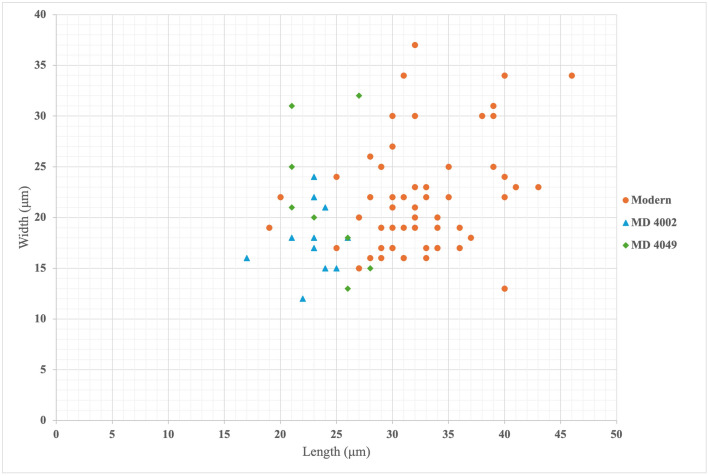
Scatterplot showing the lengths and widths of the aleurone cells observed in the maize-based experimental samples (shown in orange) and the two archaeological samples (shown in blue and green).

A scatterplot of the length and width of the aleurone cells in the experimental (N = 57) and archaeological (N = 19) samples can be seen in Fig 10. While the aleurone cells in the archaeological samples are within the size range observed in the experimental samples, they are clearly on the smaller end of the spectrum.

#### Porosity.

**Fig 11:**
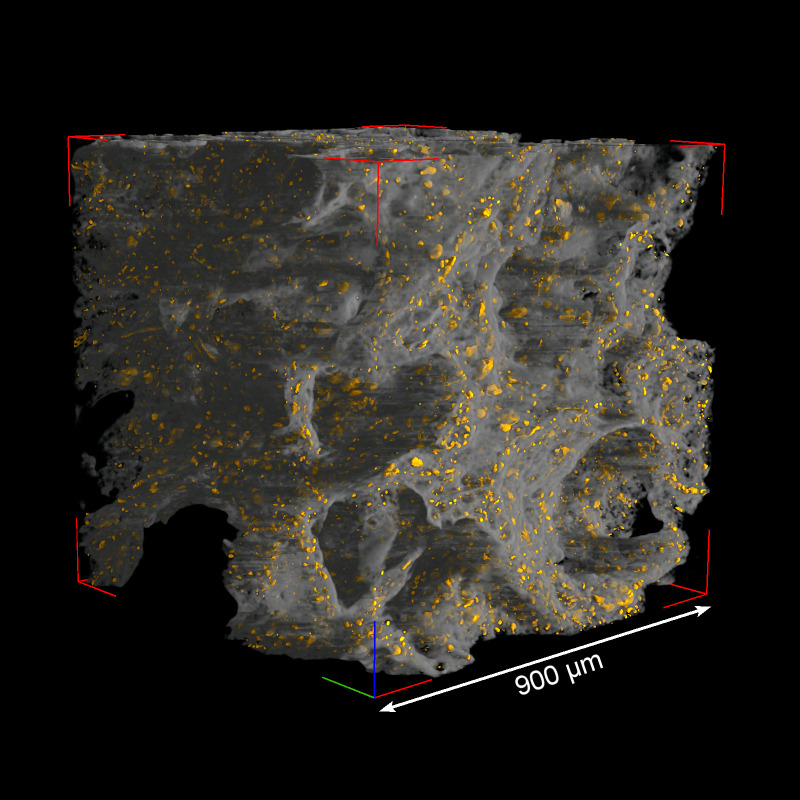
Closed porosity in the plain maize experimental sample. The solid fraction (in gray) is displayed in semi-transparency to highlight the closed porosity fraction (in yellow).

Both the experimental doughs and the archaeological samples are characterized by abundant porosity, including both closed (i.e., completely included within the solid fraction of the dough) and open (i.e., connected to the outside of the sample). In order to compare different samples, we focused on describing the closed porosity (see Fig 11; S3 Fig), as this is more easily definable, despite representing only a minor fraction of the total void space present in the considered volumes of interest (VOIs). Open porosity consists mainly of highly interconnected air-filled volumes between different branches of the dough, showing typically irregular shapes and ranging in width from approximately 50 µm to a few hundred µm; however, it also includes a fraction of smaller, more spherical voids that are partially contained within the thickness of the dough.

**Fig 12:**
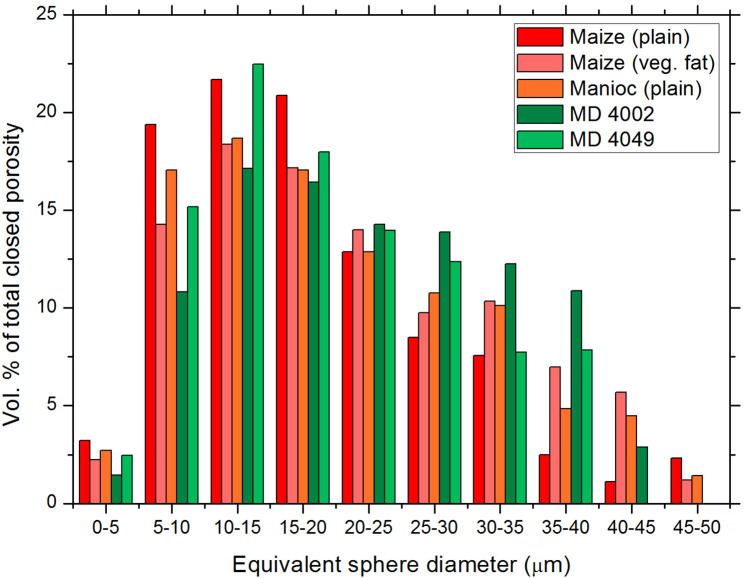
Grouped bar chart showing the size categories of the closed pores and their percentage in the various samples studied. The total number of individual pores, most of which are smaller than 5 µm, ranges from approximately 18000 to 20000 in the experimental doughs and is approximately 21000 in the archaeological samples.

The VOIs for the evaluation of the size distribution of closed pores were selected trying to avoid, as far as possible, the inclusion of plant remains such as seeds or parts of vascular tissues, in order not to include their intrinsic porosity in the calculation of the porosity of the dough itself. Cubic VOIs with a side length of 900 µm were considered for most of the samples except for the archaeological sample MD 4002, for which a more representative VOI was obtained by defining an equivalent parallelepiped with dimensions of 900 x 450 x 1800 µm^3^. The volume equivalent sphere diameter was used as a convenient parameter to describe the size of each pore and evaluate the size distribution of closed pores within the VOIs (Fig 12). In the experimental tamales, regardless of whether they are made from maize or manioc, the size of the closed pores varies approximately between 1 µm and 50 µm, with the majority of the pores (in terms of volume fraction, not absolute number) included in the size classes between 5 µm and 25 μm. The archaeological samples MD 4002 and MD 4049 are also composed of pores in these same size classes. MD 4002 seems to have a more homogeneous pore size distribution, with a less pronounced maximum between 10 µm and 20 µm and a shift towards larger sizes, while a peak of the distribution at 10–15 µm is better defined in MD 4049. The solid fraction of the doughs was characterized as well by evaluating the structure thickness parameter (mean value and dimensional distribution), calculated in the same way as the trabecular thickness in bone studies. The mean wall thickness of the dough in the experimental samples is quite similar, ranging from 14.1 µm in the maize-based tamale with added fat to 16.0 µm in the maize-based tamale without fat; an intermediate value of 15.2 µm is found in the manioc-based tamale. For the three experimental samples the maximum values of the structure thickness distribution are approximately 30–33 µm. The archaeological samples MD 4002 and MD 4049 show slightly higher values of mean wall thickness (17.9 µm and 16.6 µm, respectively) and maximum wall thickness (55–58 µm and 40–42 µm, respectively); these results are probably influenced by some localized portions of the dough characterized by very few pores, particularly in the case of MD 4002.

## Discussion

Our study demonstrates that combining SEM (which provides high-resolution external views of the fragments) with SR micro-CT can better characterize the composition of archaeological amorphous fragments. While archaeobotanists may be able to identify some seeds visible on the exterior of ACOs, SR micro-CT permits visualization of the inner architecture to determine if the sample is homogeneous, or whether it contains different structural elements belonging to potentially different plant parts.

We begin by considering the identifiable elements in the modern samples. Most notably, we demonstrated that parts of the maize kernel, such as the aleurone and the endosperm, can be visualized. The aleurone layer was clearly visible in the experimental samples, consisting for the most part of a line of rectangular cells. The aleurone layer was visible in 2D and in 3D, and was not typically observed when the samples were viewed with the SEM. This is true for both the experimental samples, but also in one of the archaeological samples. The smaller size of the aleurone cells in the archaeological samples is an issue that will have to be addressed in the future. However, some potential causal factors for this size difference include the use of different maize race varieties and different cooking processes (i.e., nixtamalization, toasting, boiling, etc.) and degree of carbonization, which typically leads to tissue shrinkage and distortion [80]. The animal bones found in the same context displayed clear burn patterns ranging from brown to black to grey/white, suggesting that some areas of Offering D1 reached heightened temperatures that would have also affected the archaeological ACOs. The recovery of aleurone cells in maize flour that has been nixtamalized is not surprising, given that this alkali-procedure does not remove the aleurone layer, instead only softening and facilitating the removal of the pericarp [81]. Although the aleurone has been shown to effectively resist carbonization [67] our study further confirms that it is a durable and characteristic feature that can be used to identify maize in ACOs, even when the kernel has undergone a number of processes that include nixtamalization, grinding, cooking, and carbonization. Therefore, we can use it as a distinguishing feature to identify the presence of cereals, as other scholars have done in different parts of the world [31–33,35]. What remains to be determined is aleurone size and eventual resistance to food processing and carbonization in other members of the grass (Poaceae) family (e.g., *Bromus*), native to the American continent. Regarding the endosperm, as demonstrated by Petrucci and Lema [67] soft and hard maize endosperms responded differently to boiling and charring. Thus, it will also be important to work with different maize races to evaluate whether endosperm differences might affect conservation after charring.

Our experimental work also proves that the recovery and identification of beans (or at least Fabaceae seeds/cotyledons) is possible in modern food remains. Pulses such as lentils and peas have been identified based on the presence of the palisade cell layer (i.e., macrosclereids) within the food remains from Neolithic Çatalhöyük [33]. Peas and *Lathyrus* have also been identified from food remains from Shanidar based on the existence of the macrosclereid layer as well as the seed coat pattern [34]. In both these cases, the seeds were likely ground and/or pounded, demonstrating the resistance of pulses to mechanical actions and to heat (carbonization). Experiments carried out using seeds from American legumes, (*Neltuma chilensis/N. flexuosa*), showed high resistance to carbonization, in particular when it came to the testa [82]. Future experiments will consider using pounded or fragmented beans as inclusions in the dough to see whether similar results can be attained.

With regards to the chia seeds, morphological characteristics that include the seed shape and size as well as the seed coat, and the presence of mucilage if the seed came into contact with water prior to being cooked or carbonized, were still present in select modern carbonized remains. The ability to visualize mucilage, even after cooking and carbonization of the sample, might provide clues as to how the chia seeds were prepared.

We demonstrated that some parts of the manioc tuber remain even after processing. With the SEM we observed xylem vessels but not the phloem. The survival of xylem in archaeological contexts has already been reported, likely because of the thick cells and non-living walls that compose xylem [83]. We did not observe any phloem in the experimental tamale, which aligns with previous observations that thinner walls are more susceptible to charring damage [21,83]. SEM also allowed us to visualize some parenchyma cells. Conversely, features were less evident when using SR micro-CT. The morphology and dimensions of the longitudinal elements suggest that they belong to the elongated cells containing parenchyma. Micro-CT imaging of parenchymatous tissues from species found in the Indo-Pacific region have revealed that it is possible to differentiate between and within species [21]. However, given that we are working with processed foods and not entire tubers, further research is needed to determine whether these features will enable us to consistently identify manioc in the archaeological record. Developing the identification of characteristics related to processed tubers native to the Americas is therefore important and needs to continue.

A first attempt on how to study and quantify the tamale dough was undertaken, and while this study is still at a preliminary stage, we can share some initial observations. Our experimental tamales are similar in their porosity, which is interesting considering their differing ingredients. Future studies will be necessary to determine to what degree the porosity is affected by factors such as temperature and exposure time to fire, as well as the role played by the starch grains present in the different flours and their physiochemical characteristics. Only then will it be possible to determine whether the porosity can be used to clearly differentiate between doughs.

The study of the archaeological sample MD 4002 revealed that some aleurone segments were present in the matrix surrounding the chia seed, one of which was still connected to the endosperm. The aleurone cells in this sample were slightly smaller than those found in the experimental ones, but they had the same rectangular shape as those in the modern samples. The chia seed associated with this matrix did not appear to have mucilage fibers. The dough/matrix differed only slightly from the experimental tamales (in terms of the size distribution of porosity and the wall thickness of the solid fraction), and when combined with the presence of the aleurones, suggests that a maize-based flour was prepared, although the exact recipe (quantities, exact ingredients, and whether maize was nixtamalized or potentially toasted or just boiled prior to grinding) currently eludes us. In summary, we can suggest that this archaeological ACO is the remains of a complex, multi-ingredient food, composed of maize dough with chia seeds, which were potentially toasted. The combination of these elements suggests that these ingredients were likely intentionally mixed (see also [32]). Sample MD 4049 is more difficult to interpret, but the presence of maize is likely given by the pericarp/testa combined with the aleurone cells seen in the inner matrix of the carbonized remain. The various fragments of the seed coat in different positions within the sample might be indicative of a fragmented carbonized maize kernel, associated with another, unidentified ingredient represented by the larger seed coat visible on the outside of the sample.

These initial experiments have demonstrated the value of combining imaging techniques on ACOs. Besides providing innovative data on the ancient foods processed by Mesoamerican cultures, they also highlight the great potential of these fragments, which are often set aside, but if properly analyzed can provide important information on past culinary practices. SR micro-CT offers a unique manner to study ACOs at a high spatial resolution when a non-destructive approach is desirable. The difficulty in observing the aleurone layer, which might be deeply embedded in an ACO, is one reason to justify a combination of imaging techniques, including those that use X-rays as they permit to see the inner part of the sample without altering it. Breaking apart these samples might indeed allow for SEM images to be taken of specific areas, but it does disrupt their integrity and prevents us from documenting the ways in which the ingredients might be interconnected. Limitations to consider include the size of the sample (which typically cannot exceed a few mm if high-resolution is required), but also limited access to a synchrotron facility (beamtime has an overall high cost and only a small number of proposals are accepted), and the complexity of developing an optimized and reliable strategy for segmenting some complex features of interest, particularly in cases where the contrast with the matrix is very low or homogeneous. Finally, the need for equipment that can process but also store large amounts of data is crucial (several TB of data can easily be acquired during one beamtime). Comparable results in terms of spatial resolution may also be obtained using the most advanced lab-based CT systems, which allow for phase-contrast imaging in some cases. However, acquisition times are much longer when compared to those achievable at a synchrotron beamline. Moving forward, we will conduct a range of other experiments, including preparing maize dough from non-nixtamalized grains, using a variety of other ingredients (consisting of different plant parts) native to the Americas, but also using controlled measures of both temperature and time. The goal is to determine whether other diagnostic plant features can be identified and utilized to better study ancient ACOs. The resulting image database will serve to provide a more nuanced understanding of ancient foods and culinary traditions, and in turn help to address issues relating to social identity construction in the past.

## Conclusions

To the best of our knowledge, this is the first study of amorphous carbonized objects originating from archaeological contexts in the New World to be analyzed using SEM and SR phase-contrast micro-CT in a complimentary manner. Through the preparation of food replicas, including tamales made from maize and manioc, with inclusions of small (chia) and larger seeds (beans), we have begun documenting the features that remain identifiable even after processes that might include grinding, cooking, and charring.

Although we are in the early stages of this research, we strongly believe that it has the potential to reveal nuances of ancient cuisine and in turn provide essential information on social identity among past cultures. One of the investigated ACOs is most probably a crumb from a complex food, composed of maize dough and chia seeds. While we cannot rule out tortillas as the origin of these carbonized crumbs, they are more likely to come from a tamale, given that these are often made with a variety of ingredients nowadays. This opens up a much broader discussion on the identity of those that produced the food. Tortillas are believed to have been produced in Teotihuacan since at least the Preclassic period given the presence of *comales*, or ceramic griddles [84]. On the other hand, the rare occurrence of *comales*, combined with iconographic and epigraphic records, and, more recently, architectural elements, indicates the importance of tamales for Classic Maya populations [48,85]. If tamales were indeed present in Offering D1, they could have been a way for the Maya to signal their participation in the feast and, more broadly, in the activities held at the Plaza of the Columns Complex. A forthcoming study of other ACOs from the same context will aim to shed more light on this argument.

Our approach, consisting of imaging at different scales, which includes OM, SEM, and SR phase-contrast micro-CT, showed that it can identify the microstructure of certain plants, and, in turn, can help to identify specific ingredients. It can also provide clues to how foods were processed. When applied to archaeological samples often labeled as “unidentified” or “unknown”, we were able to extract some tantalizing clues about how ancient foods might have been prepared. By providing new information on remains recovered during flotation, we believe that our methodology can advance the study of ancient culinary traditions in the Americas.

## Supporting information

S1 Fig SEM images of cross-sections of different maize varieties in a fresh state, popcorn (A-B) and flint (C-D), are shown at different magnifications. Anatomical details of the kernel are indicated in B and D and show the distinctive aleurone layer (A) just below the pericarp and testa (P/T), and above the starchy endosperm (SE).
(PDF)

S2 Fig Slice showing the cross-section of the bean seed coat with white spots potentially indicating calcium oxalate crystals (as shown by red arrow). (PDF)

S3 Fig Closed porosity highlighted in the various samples studied. A) Maize with vegetable fat tamale; B) manioc tamale; C) archaeological sample MD 4049; archaeological sample MD 4002. The solid fraction (in gray) is displayed in semi-transparency to highlight the closed porosity fraction (yellow color) considered for the calculation of pore size distribution.
(PDF)

S1 Table Length and width (in µm) of aleurone cells in modern tamale samples. (PDF)
