# Supplementary material for: Amorphous carbonized objects and their contribution to reconstructing ancient Mesoamerican cuisine: An innovative non-destructive methodological approach
Source: PLoS One. 2025 Nov 19;20(11):e0334457. doi: 10.1371/journal.pone.0334457 (PMC12629468; doi:10.1371/journal.pone.0334457)
Supplement: S1 Fig — Anatomical details of the kernel are indicated in B and D and show the distinctive aleurone layer (A) just below the pericarp and testa (P/T), and above the starchy endosperm (SE). (PDF) [file pone.0334457.s001.pdf]

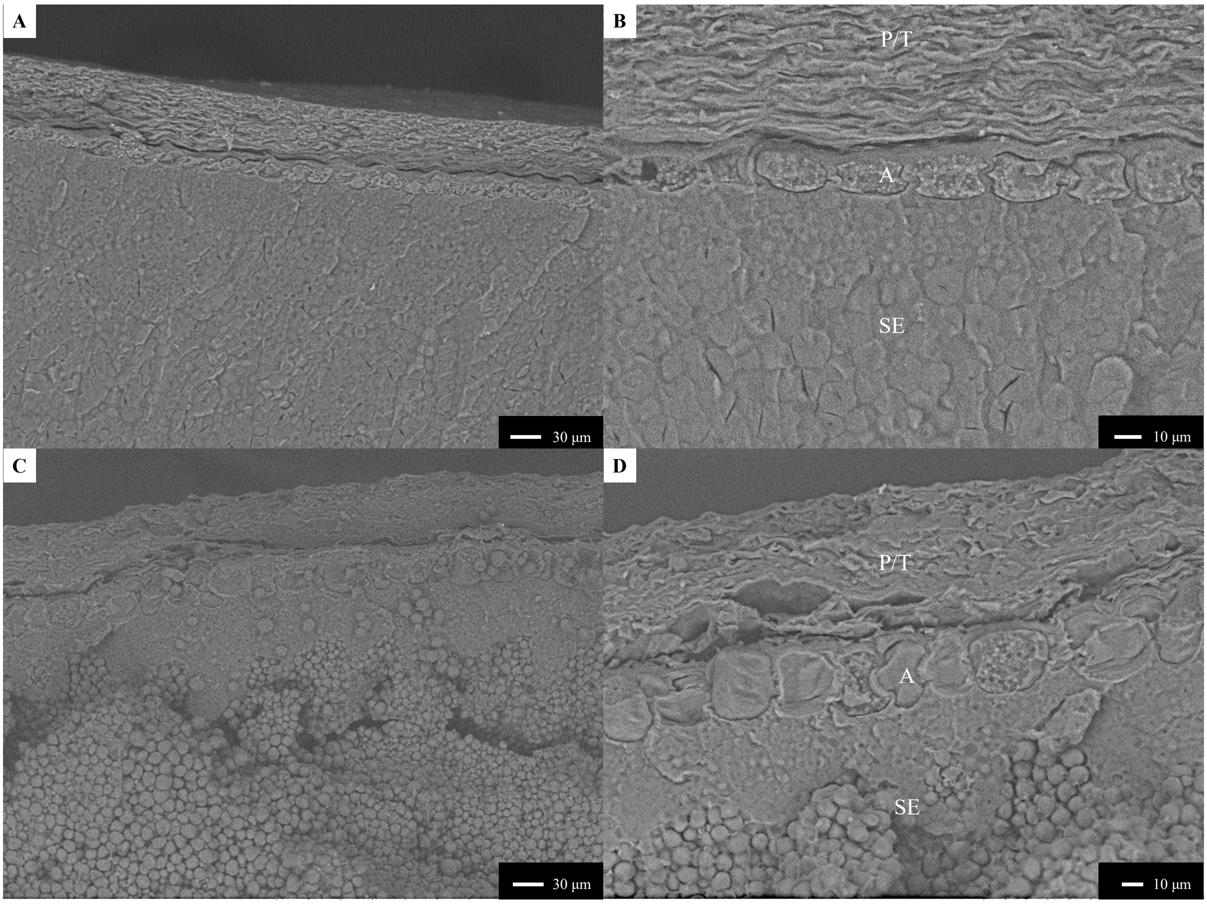

S1 Fig. SEM images of cross-sections of different maize varieties in a fresh state, popcorn (A-B) and flint (C-D), are shown at different magnifications. Anatomical details of the kernel are indicated in B and D and show the distinctive aleurone layer (A) just below the pericarp and testa (P/T), and above the starchy endosperm (SE).
