# Supplementary material for: Amorphous carbonized objects and their contribution to reconstructing ancient Mesoamerican cuisine: An innovative non-destructive methodological approach
Source: PLoS One. 2025 Nov 19;20(11):e0334457. doi: 10.1371/journal.pone.0334457 (PMC12629468; doi:10.1371/journal.pone.0334457)
Supplement: S2 Fig — (PDF) [file pone.0334457.s002.pdf]

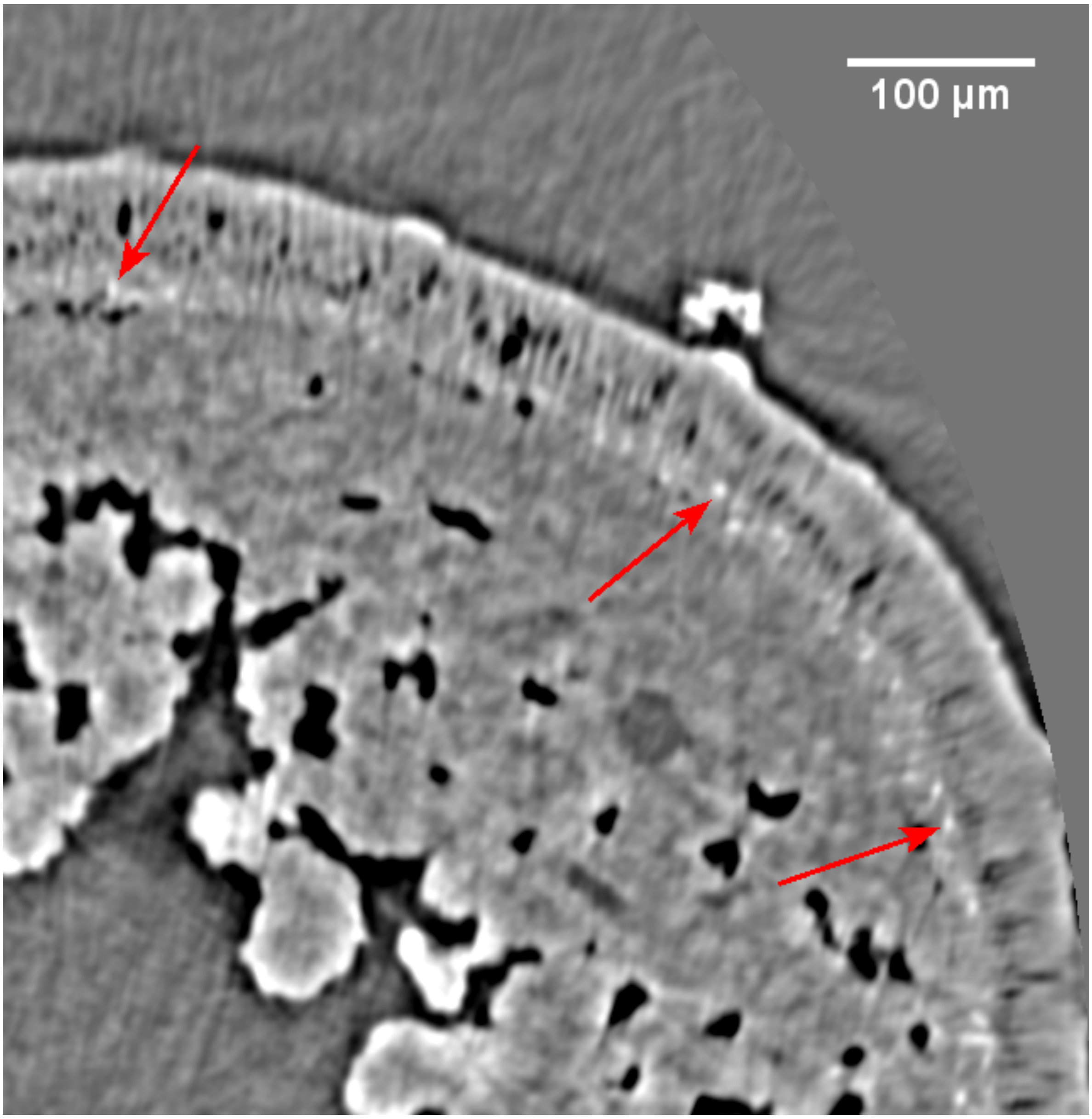

S2 Fig. Slice showing the cross-section of the bean seed coat with white spots potentially indicating calcium oxalate crystals (as shown by red arrow).
