# Supplementary material for: Amorphous carbonized objects and their contribution to reconstructing ancient Mesoamerican cuisine: An innovative non-destructive methodological approach
Source: PLoS One. 2025 Nov 19;20(11):e0334457. doi: 10.1371/journal.pone.0334457 (PMC12629468; doi:10.1371/journal.pone.0334457)
Supplement: S3 Fig — A) Maize with vegetable fat tamale; B) manioc tamale; C) archaeological sample MD 4049; archaeological sample MD 4002. The solid fraction (in gray) is displayed in semi-transparency to highlight the closed porosity fraction (yellow color) considered for the calculation of pore size distribution. (PDF) [file pone.0334457.s003.pdf]

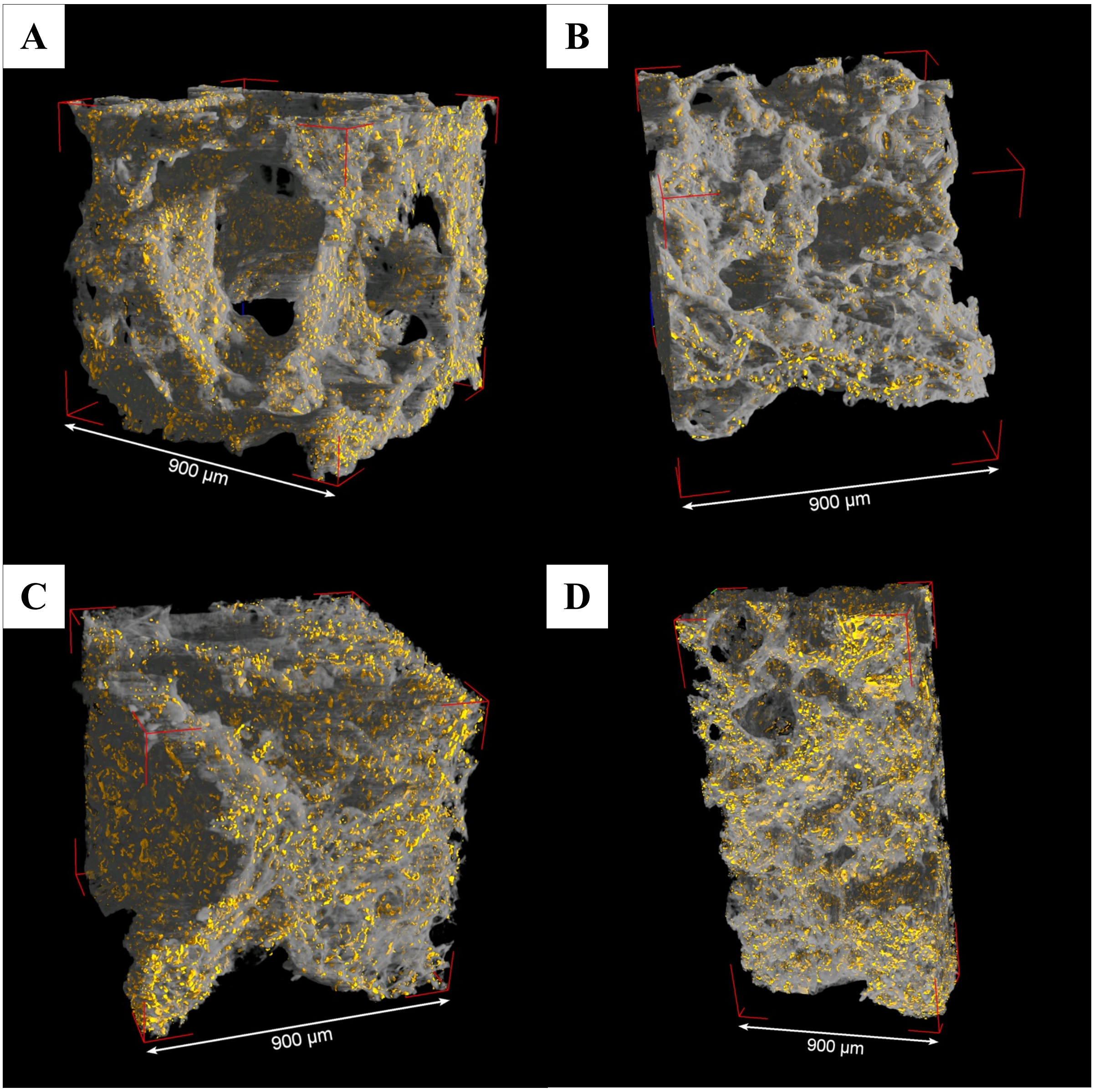

S3 Fig. Closed porosity highlighted in the various samples studied. A) Maize with vegetable fat tamale; B) manioc tamale; C) archaeological sample MD 4049; archaeological sample MD 4002. The solid fraction (in gray) is displayed in semi-transparency to highlight the closed porosity fraction (yellow color) considered for the calculation of pore size distribution.
