# Supplementary material for: Amorphous carbonized objects and their contribution to reconstructing ancient Mesoamerican cuisine: An innovative non-destructive methodological approach
Source: PLoS One. 2025 Nov 19;20(11):e0334457. doi: 10.1371/journal.pone.0334457 (PMC12629468; doi:10.1371/journal.pone.0334457)
Supplement: S1 Table — (PDF) [file pone.0334457.s004.pdf]

S1 Table. Length and width (in µm) of aleurone cells in modern tamale samples.

	Length	Width
	34	20
	33	23
	30	27
	29	17
	37	18
	30	17
	30	17
	31	22
	34	17
	30	30
	32	30
	29	25
	20	22
	25	24
	32	23
	33	17
	30	21
	33	16
	27	20
	36	19
	27	15
	32	19
	28	16
	34	17
	30	19
	30	22
	35	25
	34	19
	39	30
	43	23
	32	20
	28	26
	38	30
	32	37
	31	34
	33	22
	36	17
	41	23
	40	13
	41	23
	39	31
	40	34
	46	34
	40	24
	31	16
	31	16
	28	22
	19	19
	25	17
	31	19
	32	21
	29	19
	35	22
	39	25
	40	22
	29	25
	29	16
**Max**	46	37
**Min**	19	13
**Average**	**33**	**22**
